# Molecular identification of adenoviruses associated with respiratory infection in Egypt from 2003 to 2010

**DOI:** 10.1186/1471-2334-14-50

**Published:** 2014-01-30

**Authors:** Pola N Demian, Katherine C Horton, Adriana Kajon, Rania Siam, Amel Mohamed Nageib Hasanin, Amany Elgohary Sheta, Claire Cornelius, Anne M Gaynor

**Affiliations:** 1 U.S. Naval Medical Research Unit No. 3, Cairo, Egypt; 2 Lovelace Respiratory Research Institute, Albuquerque, NM, USA; 3 Biology Department and the YJ-Science and Technology Research Center, American University in Cairo, Cairo, Egypt; 4 Ministry of Health and Population, Cairo, Egypt; 5 Present Address: School of Veterinary Medicine, University of California, Davis, USA; 6 Present Address: 218th Medical Detachment, (Veterinary Service Support), 47th Combat Support Hospital, 62nd Medical Brigade, Joint Base Lewis-McChord, WA, USA

**Keywords:** Adenovirus, Influenza-like illness, Respiratory illness, Egypt

## Abstract

**Background:**

Human adenoviruses of species B, C, and E (HAdV-B, –C, -E) are frequent causative agents of acute respiratory infections worldwide. As part of a surveillance program aimed at identifying the etiology of influenza-like illness (ILI) in Egypt, we characterized 105 adenovirus isolates from clinical samples collected between 2003 and 2010.

**Methods:**

Identification of the isolates as HAdV was accomplished by an immunofluorescence assay (IFA) and confirmed by a set of species and type specific polymerase chain reactions (PCR).

**Results:**

Of the 105 isolates, 42% were identified as belonging to HAdV-B, 60% as HAdV–C, and 1% as HAdV-E. We identified a total of six co-infections by PCR, of which five were HAdV-B/HAdV-C co-infections, and one was a co-infection of two HAdV-C types: HAdV-5/HAdV-6. Molecular typing by PCR enabled the identification of eight genotypes of human adenoviruses; HAdV-3 (n = 22), HAdV-7 (n = 14), HAdV-11 (n = 8), HAdV-1 (n = 22), HAdV-2 (20), HAdV-5 (n = 15), HAdV-6 (n = 3) and HAdV-4 (n = 1). The most abundant species in the characterized collection of isolates was HAdV-C, which is concordant with existing data for worldwide epidemiology of HAdV respiratory infections.

**Conclusions:**

We identified three species, HAdV-B, -C and -E, among patients with ILI over the course of 7 years in Egypt, with at least eight diverse types circulating.

## Background

Human adenoviruses (HAdV) belong to the family *Adenoviridae* and the genus *Mastadenovirus*. Seven HAdV species are formally recognized by the International Committee on Taxonomy of Viruses, indicated by letters A-G and encompassing the 54 accepted HAdV types, designated by numbers 1 through 54. The clinical manifestations of HAdV infection varies with the species and type of virus, ranging from asymptomatic infection to mild respiratory (HAdV-B1, HAdV-C, HAdV-E), gastrointestinal (HAdV-F), and ocular (HAdV-B, HAdV-D, HAdV-E) disease. Life threatening pneumonia, septicemia and endocarditis are also possible in immunecompromised individuals or those with an underlying a comorbidity [1-5].

Among HAdV-associated acute respiratory disease, the majority of infections are caused by three species: HAdV-B, HAdV-C, and HAdV-E [6]. Approximately 60% of HAdV infections in children and young adults are caused by HAdV-C [7-11], and asymptomatic carriers of HAdV-C are frequently detected due to the ability of this species to establish persistent infections [7,12,13]. HAdV-B (HAdV-3, -7, -11, -14, -16, and -21) and HAdV-E (HAdV-4) are most commonly associated with outbreaks of acute febrile respiratory illness, conjunctivitis, and pneumonia in crowded populations [14-17]. Co-infections with multiple types, either from the same species or different species, have been shown to occur frequently in immunocompromised patients [18,19] and have been documented in other susceptible populations [20,21].

Compared to other regions of the globe, relatively few studies have examined the contribution of HAdV to the burden of respiratory, ocular, or enteric disease in Egypt [6,22-26]. Only one study was conducted in Egypt to determine the most prevalent species and types of HAdV-associated influenza-like illness (ILI) [6]. In the current study, we used a molecular typing approach to characterize HAdV species and types associated with ILI from eight sites across Egypt from 2003–2010.

## Methods

### Sample collection

**Figure 1:**
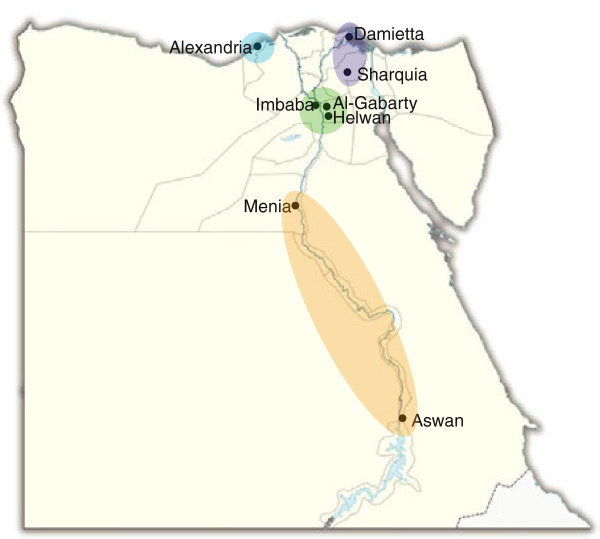
**Map displaying locations of 8 surveillance sites through Egypt, grouped by region – Alexandria (blue), Delta Region (purple), Greater Cairo (green), and Upper Egypt (orange).** The number of isolates by species and type for each region is displayed in Table 1.

Adenovirus isolates (n = 105) examined in this study were derived from oropharyngeal (OP) swabs (n = 99) obtained from patients that presented to one of eight surveillance sites from 2003–2010 (Figure 1). All samples were obtained from pediatric and adult patients that met the World Health Organization (WHO) ILI surveillance case definition (fever >38°C and respiratory manifestations of cough, sore throat or coryza with onset of illness within the previous 3 days) and were enrolled. OP swabs were obtained along with basic demographic information. OP swabs were placed in viral transport medium (VTM) and transported to NAMRU-3 in Cairo, Egypt, where they were stored at -70°C until further processing.

### Ethical approval

Ethical approval was obtained from the institutional review board (IRB) of the US Naval Medical Research Unit No. 3 (NAMRU-3), protocol DOD#NAMRU3.2004.0023 (N3 169), in compliance with all applicable federal regulations governing the protection of human subjects. Written informed consent was waived by the IRB, and samples contained no patient identifiers or links to clinical data. The protocol, including the exemption of written informed consent, received signatory approval from the Egyptian Ministry of Health and Population prior to IRB approval.

### Virus isolation

Clinical specimens were treated with 10 μl of each of the following antibiotics: penicillin (100,000U/ml)/streptomycin (100,000 μg/ml), gentamicin (50 mg/ml)) and amphotericin B (2.50 μg/ml) as an anti-fungal agent, for ten minutes. 100 μl of each sample was inoculated onto three cell lines: MDCK (ATCC# CCL-34), NCI-H292 (ATCC# CRL-1848) and LLC-MK2 (ATCC# CCL-7.1) to maximize recovery of respiratory viral pathogens. Cell cultures were examined for cytopathic effect (CPE) for 12–14 days post-inoculation. One hundred and five HAdV isolates were recovered. HAdV identification was determined by indirect immunoflourescence antibody (IFA) test (Respiratory Viral Screen IFA Kit, LIGHT DIAGNOSTICS™, Millipore, MA, USA). After confirmation, infected tissue culture fluid (ITCF) was collected and stored at -70°C until further processing.

### DNA extraction

DNA was extracted from 200 μl of ITCF using MagNA Pure LC Total Nucleic Acid Isolation Kit (Roche). Extracted DNA was stored at -20°C for further analysis.

### Control strains for adenovirus

The prototype strains of HAdV were obtained from American Type Culture Collection (ATCC): HAdV-1 (VR-1), -2 (VR-846), -3 (VR-3), -4 (VR-1572), -5 (VR-5), -6 (VR-6), -7 (VR-7), -11 (VR-12), -14 (VR-15), and -21 (VR-256) to be used as controls.

### Molecular identification of adenovirus species and genotype

Primers used for adenovirus identification and species determination were obtained from the literature. For PCR confirmation of HAdV IFA positives, we used the universal primers designed by Xu et al. [27] and followed their protocol and cycling conditions with the exception of using a 25 μl reaction instead of the 50 μl used in the paper. Determination of viral species was accomplished by a minor modification of the BCE Multiplex [28] by utilizing the HadV-B reaction as a monoplex and maintaining a duplex reaction for HAdV-C and HAdV-E. This multiplex is able to discriminate between HAdV4a-like and HAdV-4p-like based on a 30 bp insertion/deletion mutation in the E1A gene, as described previously [11]. For further PCR-based typing of HAdV-B positive samples, multiplex PCR for HAdV-3, 7 and 21 [29] was performed, and any negative samples were then tested with specific assays for HAdV-11, 14, 16, and 35, as previously described [6]. Typing of the HAdV-C species was performed by a multiplex PCR targeting the fiber gene [30]. All PCR reactions were performed as described in their respective publications. All products were run on a 2% agarose gel at 100 V with ethidium bromide and were visualized and recorded by photography on a UV light box.

### Data analysis

Data were analyzed using SAS software, version 9, of the SAS System for Windows (SAS Institute Inc). Chi-square goodness of fit test was used to assess significance of bivariate associations. When expected values did not meet the frequency assumptions required for this test, Fisher’s exact test was used. Associations were considered significant by a p-value less than 0.05.

## Results

**Figure 2:**
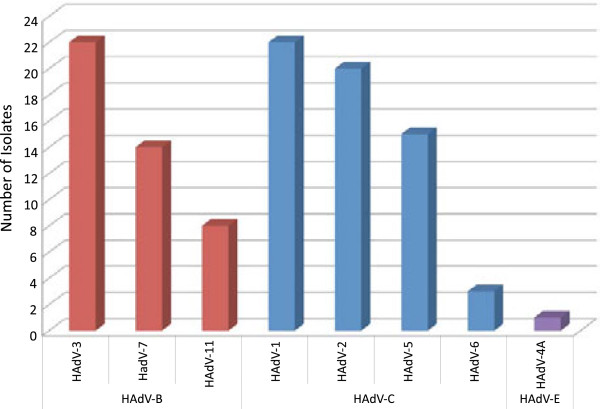
Distribution of adenovirus positive isolates (n = 105), by species – HAdV-B (red), HAdV-C (blue), HAdV-E (purple) – and type.

In this study, we sought to characterize adenovirus isolates with the aim of identifying the most prevalent species and types circulating in Egypt in recent years. OP swabs from patients with ILI were collected during the period 2003–2010. The samples were tested for the presence of a viral etiology by inoculation onto cell monolayer and the observed for CPE. Samples showing CPE were then screened for HAdV by an immunofluorescence assay and confirmed with our PCR testing algorithm. A total of 105 HAdV isolates were identified among the 99 HAdV-positive samples; 44 (42%) were HAdV-B, 60 (57%) were HAdV-C, and 1 (1%) was HAdV-E (Table 1, Figure 2). Co-infections were detected in 6 (6%) of the 99 specimens, 5 (5%) with HAdV-B and HAdV-C and 1 (1%) with two different types of HAdV-C. Molecular typing of HAdV-B species (Figure 2) identified the following types: HAdV-3 (n = 22), HAdV-7 (n = 14), and HAdV-11 (n = 8) (p = 0.03). Among the HAdV-C isolates, we identified significantly more HAdV-1 (n = 22) and HAdV-2 (n = 20) than HAdV-5 (n = 15) and HAdV-6 (n = 3) (p < 0.01). One sample in this group contained a coinfection of HAdV-5/HAdV-6. The five HAdV-B/HAdV-C coinfections included HAdV-3/HAdV-1 (n = 3), HAdV-3/HAdV-2 (n = 1), and HAdV-3/HAdV-5 (n = 1). The only HAdV-E isolate was typed as a HAdV-4a-like genomic variant.

**Table 1:** Descriptive data, by year, region, age and sex, for HAdV infections by species and genotype (n = 105)

	**HAdV-B**								**HAdV-C**										**HAdV-E**				**TOTAL**
	**HAdV-3**		**HAdV-7**		**HAdV-11**		**Total**		**HAdV-1**		**HAdV-2**		**HAdV-5**		**HAdV-6**		**Total**		**HAdV-4**		**Total**		
	**n**	**%**	**n**	**%**	**n**	**%**	**n**	**%**	**n**	**%**	**n**	**%**	**n**	**%**	**n**	**%**	**n**	**%**	**n**	**%**	**n**	**%**	
**Year**																							
2003	0	0%	0	0%	0	0%	0	0%	1	50%	0	0%	0	0%	1	50%	2	100%	0	0%	0	0%	2
2004	0	0%	0	0%	0	0%	0	0%	0	0%	0	0%	0	0%	0	0%	0	0%	0	0%	0	0%	0
2005	2	40%	0	0%	2	40%	4	80%	0	0%	0	0%	1	20%	0	0%	1	20%	0	0%	0	0%	5
2006	3	38%	0	0%	0	0%	3	38%	2	25%	2	25%	1	13%	0	0%	5	63%	0	0%	0	0%	8
2007	2	20%	0	0%	1	10%	3	30%	3	30%	1	10%	3	30%	0	0%	7	70%	0	0%	0	0%	10
2008	3	30%	4	40%	1	10%	8	80%	0	0%	2	20%	0	0%	0	0%	2	20%	0	0%	0	0%	10
2009	9	29%	5	16%	1	3%	15	48%	6	19%	5	16%c	4	13%	0	0%	15	48%	1	3%	1	3%	31
2010	3	8%	5	13%	3	8%	11	28%	10	26%	10	26%	6	15%	2	5%	28	28	0	0%	0	0%	39
**Region**																							
Alexandria	8	33%	0	0%	0	0%	8	33%	7	29%	5	21%	2	8%	1	4%	15	63%	1	4%	1	4%	24
Delta	2	22%	3	33%	1	11%	6	67%c	3	33%	0	0%	0	0%	0	0%	3	33%	0	0%	0	0%	9
Greater Cairo	8	19%	8	19%	7	17%	23	55%	5	12%	6	14%	7	17%	1	2%	19	45%	0	0%	0	0%	42
Upper Egypt	4	13%	3	10%	0	0%	7	23%	7	23%	9	30%	6	20%	1	3%	23	77%	0	0%	0	0%	30
**Age**																							
< 2 years	3	8%	5	14%	0	0%	8	22%	8	22%	8	22%	10	28%	1	3%	27	75%	1	3%	1	3%	36
2-17 years	11	23%	9	19%	7	15%	27	56%	7	15%	9	19%	3	6%	2	4%	21	44%	0	0%	0	0%	48
≥ 18 years	7	37%	0	0%	1	5%	8	42%	6	32%	3	16%	2	11%	0	0%	11	58%	0	0%	0%	0%	19
Unknown	1	50%	0	0%	0	0%	1	50%	1	50%	0	0%	0	0%	0	0%	1	50%	0	0%	0	0%	2
**Sex**																							
Male	7	17%	8	19%	4	10%	19	45%	7	17%	9	21%	5	12%	2	5%	23	55%	0	0%	0	0%	42
Female	14	23%	6	10%	4	7%	24	39%	14	23%	11	18%	10	16%	1	2%	36	59%	1	2%	1	2%	61
Unknown	1	50%	0	0%	0	0%	1	50%	1	50%	0	0%	0	0%	0	0%	1	50%	0	0%	0	0%	2
**TOTAL**	22	21%	14	13%	8	8%	44	42%	22	21%	20	19%	15	14%	3	3%	60	57%	1	1%	1	1%	105

### Distribution of HAdV species among age groups

**Figure 3:**
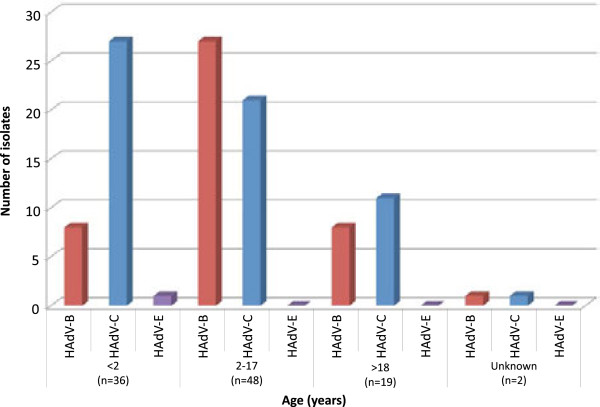
Distribution of adenovirus positive isolates by age-group and species (n = 105) – HAdV-B (red), HAdV-C (blue), HAdV-E (purple).

The age range of patients from whom HAdV isolates were obtained was 2 months to 56 years. Thirty-six of the 105 HAdV isolates were obtained from children under two years of age (Table 1, Figure 3). Of those, HAdV-C (n = 27) was more common than HAdV-B (n = 8) or HAdV-E (n = 1) (p < 0.01). Three (9%) samples had HAdV-B/HAdV-C coinfections and 1 (3%) had a HAdV-C coinfection. Forty-eight isolates were recovered from children ages 2 through 17 years. Of these, there was no statistical difference in serotypes (p = 0.12) or coinfections identified. In the third age group, 18 years of age and older, 19 isolates were recovered, including one HAdV-B/HAdV-C coinfection, again with no significant differences (p = 0.81). Age is unknown for one of the HAdV-B/HAdV-C coinfections. Across the entire patient population, HAdV-B was significantly more common among ages 2 through 17 (p < 0.01) and HAdV-C was significantly more common in ages less than 2 (p < 0.01). Coinfections were significantly more likely in patients under age 2 than in older patients (p = 0.04).

### Geographic distribution of HAdV species

Clinical specimens were obtained at eight surveillance sites distributed throughout 4 major regions in Egypt (Table 1, Figure 1). In Alexandria, HAdV-C was relatively more prevalent than HAdV-B (p < 0.01). The only HAdV-E strain was also isolated from this location. Samples collected in Upper Egypt were significantly more likely to be HAdV-C (n = 23) than HAdV-B (n = 7) (p < 0.01). There were no statistically significant differences in species proportions in the Delta region (p = 0.32) or in Greater Cairo (p = 0.54).

### Distribution of species by year of isolation and season

Significantly more isolates, 59%, were recovered from samples collected from March-June, which represents the spring and beginning of summer when average temperatures increase from 15°C in February to between 17°C and 27°C, than in the rest of the year (p < 0.01) (Table 2). Only 28% of isolates were recovered from samples during the typical influenza virus season (September-February). From the beginning of surveillance, none of the identified types circulated continuously during the eight years of sampling. HAdV-3 (HAdV-B) and HAdV-5 (HAdV-C) were detected every year after first being identified in 2005 (Table 2). HAdV-2 appeared in 2006, and HAdV-7 in 2008, and both circulated through 2010. The other types were identified sporadically throughout the surveillance period. The only HAdV-E isolate was recovered in April 2009.

**Table 2:** Distribution of HAdV infections by month and year (n = 105)

	**Jan**	**Feb**	**Mar**	**Apr**	**May**	**Jun**	**Jul**	**Aug**	**Sep**	**Oct**	**Nov**	**Dec**
**2003**	0	0	0	2	0	0	0	0	0	0	0	0
**2004**	0	0	0	0	0	0	0	0	0	0	0	0
**2005**	0	0	0	0	0	1	0	2	0	2	0	0
**2006**	0	0	1	0	0	0	1	0	1	0	3	2
**2007**	1	2	1	0	0	6	0	0	0	0	0	0
**2008**	0	0	0	0	2	0	1	3	0	2	1	1
**2009**	1	2	8	5	3	4	1	0	0	3	2	2
**2010**	0	4	5	7	9	8	4	2	0	0	0	0
**TOTAL**	**2**	**8**	**15**	**14**	**14**	**19**	**7**	**7**	**1**	**7**	**6**	**5**

## Discussion

In this retrospective study, we characterized HAdV isolates derived from an ILI surveillance program conducted as collaboration between the Egyptian Ministry of Health and Population and NAMRU-3 between 2003 and 2010. This study builds on knowledge gleaned from previous work conducted by Metzgar et al. that examined isolates in the NAMRU-3 collection from 1999 to 2002 [6].

Data from the current study indicate that HAdV continues to circulate among outpatients with ILI in Egypt and that HAdV-B and HAdV-C appear to remain the most prevalent species within this patient population. In contrast to the earlier results reported by Metzgar et al., HAdV-C was recovered in a higher prevalence than HAdV-B [6]. Though not statistically significant (p = 0.11), this relative prevalence is consistent with findings in Malaysia [7], though differs from studies in the United Kingdom [17] and Korea [31]. Unfortunately additional data from elsewhere in the region are not available for comparison. Differences in dominant HAdV species could be due to factors such as variations in geographic location, time period and sampling methodologies. Additionally, studies also indicate that HAdV-C is the most prevalent HAdV recovered from upper respiratory tract infections, in agreement with our findings from outpatients with ILI [32,33], whereas HAdV-B1 serotypes (HAdV-3, -7) are more prominent in lower respiratory tract infections [28,34-37]. Another possible explanation is that we may have detected both active and latent infections of HAdV-C through use of OP swabs [38], since laboratory methods used do not allow us to differentiate the two [28,32,34,37,39].

Among the HAdV-B isolates recovered, significantly more cases of HAdV-3 and HAdV-7, the primary HAdV-B types in respiratory infections, were identified, both of which have been associated with severe outbreaks and are classified in subspecies HAdV-B1 [6]. Contrastingly, infection with HAdV-11 (subspecies HAdV-B2) appears rare, especially in respiratory infections [40]. HAdV-1 was the most common HAdV-C type identified, followed by HAdV-2 and HAdV-5. Only two HAdV-6 isolates were identified in our collection of HAdV isolates, and this type was not detected in the previous study in Egypt [6]. Comparable proportions of HAdV-1, -2 and -5 were identified by Garnett et al., though this distribution is unexpected since HAdV-1 and -2 are more generally common in respiratory infections than HAdV-5 [38]. The HAdV-E isolate was typed as HAdV-4, the sole human type in this species, which is relatively rare in civilian populations, though associated with viral conjunctivitis outbreaks in Japan [41] and Australia [42]. It is more commonly associated with high rates of febrile respiratory illness in US military recruits [6,43-45]. The single HAdV-4 isolate was identified in April, which preceded the introduction of influenza A (H1N1) pdm09 into Egypt in June 2009 [46,47].

HAdV-B and HAdV-C infections were significantly associated with younger patients, which is expected given that HAdV is the second most common viral respiratory pathogen in children under 2 years of age [48]. HAdV-C was strongly associated with age less than 2, which may be related to the fact that this species is often endemic in this age group [6] and that the presence of HAdV-C in the tonsils peaks at age 4 [38]. Although HAdV-E is typically associated with adult infection [32], the only case observed in this study occurred in a child under two years of age. Adenoviral coinfections were also more frequent among patients under age 2 (p = 0.04). It should be noted that this study might have under-estimated the number of coinfections due to the testing algorithm that was utilized. The initial isolation was performed in cell lines routinely used in our laboratory for recovery of all respiratory viruses. However, given the differences in known cell surface receptors for HAdV types, we could have biased our isolation of certain species and types, and therefore also coinfections within a given sample. The PCR protocol for HAdV-B typing could have also led to under-estimation. Samples that were HAdV-B positive, but negative for the HAdV-3/7/21 multiplex were tested for the remainder of the HAdV-B types, HAdV-11, -14, -16, and -35, but samples that were positive for HAdV-3/7/21 were not tested for the additional types, and we could have missed samples that contained a coinfection of multiple types of HAdV-B.

HAdV occurs throughout the year, with outbreaks that are common from late winter through early summer months [5]. Although denominator data to support examination of seasonality are not available, it is notable that 58 out of the 105isolates identified in this study were isolated over a four-month period from March through June (Table 2), suggesting that the peak of circulation occurred during this time frame. This timing is also consistent with the expected circulation of HAdV-C [32], which was the most frequent species observed in this study. Larger sample sizes and broader surveillance data on ILI are required to fully delineate the seasonal distribution of HAdV.

Human adenovirus infections are a significant cause of a wide range of disease, including respiratory infections [28,33,49], and this study has established that HAdV continues to circulate in Egypt. The dominant species in circulation appears to be HAdV-C, though HAdV-B and HAdV-E have also been identified in patients with ILI. The circulation of HAdV-3, HAdV-5 and HAdV-7 is of note as these types are recognized causes of more severe acute respiratory infection [50]. Scientists and medical personnel concerned with the etiology of acute respiratory infections in Egypt should consider HAdV as a potential cause, given this evidence of infection among patients with ILI.

## Conclusions

We identified three species, HAdV-B, -C and -E, among patients with ILI over the course of 7 years in Egypt, with at least eight diverse types circulating. Continued surveillance and characterization is necessary to understand the contribution of HAdV and its genotypes to acute respiratory disease in the region.

## Abbreviations

ATCC: American Type Culture Collection; CPE: Cytopathic effect; DNA: Deoxyribonucleic acid; HAdV: Human adenovirus; IFA: Indirect immunofluorescence assay; ILI: Influenza-like illness; ITCF: Infected tissue culture fluid; LLC-MK2: Rhesus Monkey Kidney Epithelial Cells; MDCK: Madin Darby Canine Kidney; NAMRU-3: US Naval Medical Research Unit-3; NCI-H292: National Cancer Institute: Human mucoepidermoid pulmonary carcinoma; OP: Oropharyngeal; PCR: Polymerase chain reaction; VTM: Viral transport media.

## Competing interests

The authors declare that they have no competing interests.

## Authors’ contributions

PD: sample testing, confirmation, data analysis, and drafting and revising of manuscript. KH: data analysis and drafting and revising of manuscript. AK: confirmation and additional characterization, data analysis, and drafting and revising of manuscript. RS: mentoring of work and development of manuscript. AH: supervision of surveillance, advice and revision of manuscript. AS: supervision of surveillance, advice and revision of manuscript. CC: project supervision, data analysis, development and drafting of manuscript. AG: project supervision, data analysis, drafting and revising of manuscript. All authors read and approved the final manuscript.

## Pre-publication history

The pre-publication history for this paper can be accessed here:

http://www.biomedcentral.com/1471-2334/14/50/prepub

## Disclaimers

This study was supported by the governments of Egypt and the United States. The views expressed in this article are those of the authors and do not necessarily reflect the official policy or position of the Department of the Navy, Department of Defense, the U.S. Governments or the Egyptian Ministry of Health and Population. Work was funded by work unit # 847705.82000.25GB.E0018. Authors PD, KH, CC and AG are employees of the U.S. Government. This work was prepared as part of their official duties. The work of A.E.K. was funded by the Global Emerging Infections Surveillance and Response System, a Division of the US Armed Forces Health Surveillance Center. Title 17 U.S.C. §105 provides that ‘Copyright protection under this title is not available for any work of the United States Government.’ Title 17 U.S.C. §101 defines a U.S. Government work as a work prepared by a military service member or employee of the U.S. Government as part of that person’s official duties.
